# Development of Monoclonal Antibodies in China: Overview and Prospects

**DOI:** 10.1155/2015/168935

**Published:** 2015-02-25

**Authors:** Mao-Yu Zhang, Jin-Jian Lu, Liang Wang, Zi-Chao Gao, Hao Hu, Carolina Oi Lam Ung, Yi-Tao Wang

**Affiliations:** State Key Laboratory of Quality Research in Chinese Medicine, Institute of Chinese Medical Sciences, University of Macau, Macau

## Abstract

Monoclonal antibodies (mAbs) have become increasingly important as human therapeutic agents. Yet, current research concentrates on technology itself and pays attention to developed countries. This paper aims to provide a comprehensive review of mAbs development in China through systematic analysis of drug registry, patent applications, clinical trials, academic publication, and ongoing R&D projects. The trends in therapeutic areas and industrialization process are also highlighted. Development and research trends of mAbs are analyzed to provide a future perspective of mAbs as therapeutic agents in China.

## 1. Introduction

**Figure 1:**
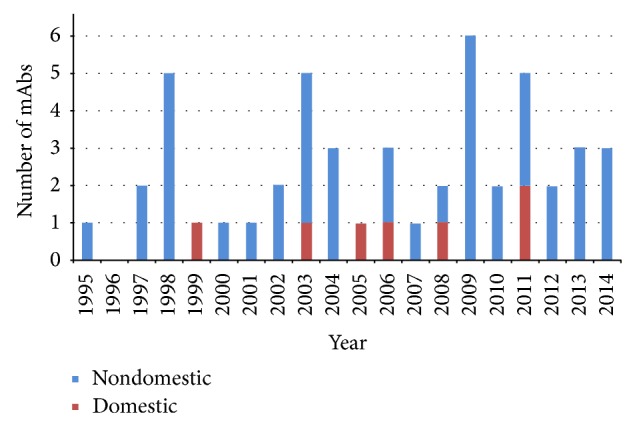
The development progress of antibody technology and antibody drug (date current as December 2014). Notes: 2014: Ramucirumab (VEGFR-2), Siltuximab (IL6), and Vedolizumab (*α*4*β*7). 2013: Itolizumab (CD6) [Ind], Ado-trastuzumab emtansine (HER2), and Obinutuzumab [CD20]. 2012: Pertuzumab (HER2). 2011: Belimumab (BLyS), Ipilimumab (CTLA-4), and Brentuximab (CD30), (CD25) ^*^, and (TNF) ^*^. 2010: Tocilizumab (IL6R) and Denosumab (RANK-L). 2009: Golimumab (TNR), Canakinumab (ILIb), Catumaxomab (EPCAM/CD3), Ustekinumab (IL12/23), and Ofatumumab (CD20). 2008: Certolizumab (TNF), (EGFR) ^*^. 2007: Eculizumab (CD5). 2006: Ranibizumab (VEGF), Panitumumab (EGFR), and (HA18G/CD147) ^*^. 2005: (TNF) ^*^. 2004: Cetuximab (EGFR), Bevacizumab (VEGF), and Natalizumab (*α*-4integrin). 2003: I-Tositumomab (CD20), Omalizumab (IgE), Efalizumab (CdIIa), Alefacept (CD2), and (IL-8) ^*^. 2002: Adalimumab (TNF) and In/90-Ibritumomab (CD20). 2001: Alemtuzumab (CD52). 2000: Gemtuzumab (CD33). 1999: (CD3) ^*^. 1998: Basiliximab (IL2R), Palivizumab (RSV), Infliximab (TNF), Trastuzumab (HER2), and Etanercept (TNF2). 1997: Rituximab (CD20) and Daclizumab (IL2R). 1996: N/A. 1995: Edrecolomab (EPCAM).  ^*^Domestic mAb product of China.

Over the past three decades, monoclonal antibodies (mAbs) have achieved a dramatic development from scientific tools to powerful human therapeutic agents [1] (see Figure 1). Sales of mAbs therapies exceeded 40 billion US dollars in 2010 and are expected to reach 70 billion US dollars by 2015 [2]. In 1975, Kohler and Milstein firstly described the* in vitro* production of murine mAbs from hybridomas [3], which was an innovative step towards the development of human mAbs as therapeutic agents. In the late 1980s, clinical development of murine mAbs was initiated but then inhibited by numerous significant drawbacks [4]. Later, in attempt to overcome the inherent immunogenicity concerns and reduce effector function of murine mAbs in human [5], chimeric mouse-human antibodies were developed [6, 7]. Then, humanization antibodies were developed [8], which significantly enlarged the clinical usage of mAb. In the following research exploration, human antibodies developed by phage display [9–11] and human Ig mice advanced the development of mAb greatly [12, 13]. Nowadays, human mAbs are the fastest growing category of mAb therapeutics entering clinical study. Development of this class of therapeutic agents started as early as 1980s but achieved no clinical or commercial success until 2002 when adalimumab became the first human mAb approved by the US Food and Drug Administration (FDA) [14].

Clinical development of mAbs in China, like many developed countries, started with murine mAb [15]. R&D of mAbs in China began in the 1980s [16] and the first mAb therapeutic agent (Murine Monoclonal Antibody against Human CD3 Antigen of T Lymphocyte for Injection) was introduced in 1999 [17]. Although mAbs development in China has made significant progress over the past 2 decades [18], all the mABs currently approved by CFDA are technologically outsourced from foreign firms, like Avastin (Roche) [19]. These mAbs mainly target CD20 [20–22], antitumor necrosis factor (TNF) *α* [23, 24], VEGFR [25–27], HER 2 [28, 29], and EGFR [30, 31] for the treatment of cancer or immunological disorders [32]. Recently, 131I-chTNF and humanized mAb h-R3 have been developed and approved as the treatment for solid tumor after Panorex [16]. It is anticipated that continuous research on mAbs will give rise to an expanding source of therapeutics.

Stepping into 21st century, the technology level of mAbs in China has vastly improved, resulting in great research progress resulting in high expression and specificity. For example, mAbs based on research of TNF*α* were developed to treat rheumatoid arthritis (RA) for patients presenting with medium and severe symptoms [33]. These mAbs block the interaction between VEGF and KDR molecule and exhibit high specificity and activity [34].

This paper aims to provide a comprehensive review of mAb development in China through systematic analysis of product registry, patent application, clinical trials, academic publication, and ongoing R&D projects.

## 2. Method

We used multiple sources for data collection. All mAbs products approved for marketing in China were searched in the drug registry maintained by CFDA. The United States Patent and Trademark Office (USPTO) was the data source for the analysis of patent applications submitted by Chinese assignee. All clinical trials involving mAbs approved and carried out in China were identified at the Chinese Clinical Trial Registry (ChiCTR) database. Academic publications about mAbs published by researchers in mainland China were searched from Thomson Reuters' Web of Science (WOS). Information about the progress of the ongoing R&D mAbs projects was collected from the main research institutes and firms in China. In this research, we focused primarily on the mAbs development in mainland China and thus the corresponding data about Hong Kong, Taiwan, or Macao was not considered. Based on the information from the abovementioned sources, this paper tries to present a comprehensive analysis of mAbs development in China which will help provide some perspectives for continuous development of these therapeutic agents.

## 3. mAbs in China

### 3.1. Registry of Domestic mAbs Products in China

Murine Monoclonal Antibody against Human CD3 Antigen of T Lymphocyte for Injection was the only mAb approved by CFDA since 1999 until 2003 when CFDA granted approval to the second mAb. Up to date, there are a total of eight mAb products approved by CFDA (see Table 1).

#### 3.1.1. Clinical Indications

Most of the mAbs approved as human therapeutic agents are approved for the treatment of cancer or immunological disorders [6, 35]. Of the eight mAbs launched in China, three target antigens related to antineoplastic diseases, five target antigens related to immunological diseases, and two target antigens related to kidney transplant rejection.

As shown in Table 1, there are three products approved for oncological uses including stage III/IV of nasopharyngeal carcinoma (NPC), advanced liver cancer, and non-small cell lung cancer (NSCLC). Another three products are indicated for immunological diseases, namely, psoriasis vulgaris, subacute eczema, active rheumatoid arthritis, moderate and severe plaque psoriasis, and ankylosing spondylitis. There are also products which are used to prevent rejection after organ transplantation.

#### 3.1.2. Molecular Targets

The target of a therapeutic antibody is a major determinant of its efficacy and safety profile [14]. The eight mAbs products which are approved by CFDA target a total of seven unique antigens. These include CD147, EGFR, intracellular DNA for tumor therapy, IL-8, TNF for immunological diseases, and CD 3 and CD25 for prevention of organ rejection after transplantation. There is also one mAb which target the nucleus of tumor cells.

In terms of versions, among the eight mAbs approved in China, three are murine, one is chimeric, two are humanized and two are recombinant fusion protein. At present, there is no fully human mAb approved for marketing in China.

#### 3.1.3. Technology Source

The development of mAb technology remains at an early stage in China. Overall, the mAbs being studied are “me-too” and “me-better” products acquired through internal and external cooperation. On one hand, drug companies and universities with sufficient capacity conducted joint R&D projects. On the other hand, mAb producers in China directly purchased the production technology from foreign countries, as demonstrated by the technology purchase actions of Shanghai Mei'en (collaborate with University of Southern California, USA) and Shanghai Celgen (transfer from Condar Co., Ltd., USA).

### 3.2. Patent Application about Chinese mAbs

According to the USPTO database, the first mAb-related patent in China was granted to monoclonal antibody against hepatitis E virus or its fragment with binding activity and use thereof in 2006. Notably, there are a total of nine mAb-related patents approved up to date.

Of these nine patents (see Table 2), three are related to cancer diagnosis and therapy along with four other mAbs targeting TNF, EFGR, and VEGFR. Two patents which are associated with infectious diseases focus on virus or its fragment with binding activity and use thereof. For patent assignee, academic institutions are the most common and powerful controller in China, such as the Institute of Basic Medical Sciences of Chinese Academy of Medical Sciences, Shanghai Cancer Institute, and Xiamen University.

### 3.3. Clinical Trials of mAbs Products in China

Data of clinical trial registry indicates that there are twelve mAbs products currently undergoing clinical studies in China which have been scheduled to complete by the end of 2013.

#### 3.3.1. Study Phase

The stages of clinical studies for the 12 mAbs were analyzed as shown in Table 3. Four candidates are in Phase II (bevacizumab, bimotuzumab and cetuximab), three in Phase III (rituximab, infliximab and bevacizumab), and five under postmarketing surveillance (ranibizumab, metuximab and rituximab). Among these, seven were chimeric mAbs while the other five were humanized mAbs.

#### 3.3.2. Targets and Indications

Among these twelve mAb candidates, eight are studied as the treatment for cancer while the rest are tested for their use in ophthalmology and immunology. Three of the eight mAbs for cancer treatment are under review for market reassessment. The technology to develop mAbs as antitumor drugs is considered mature. For example, as the first mAb product used in non-Hodgkin's lymphoma (NHL) therapy, Rituxan has demonstrated great achievements in both clinical setting and market share once it has been approved. mAbs for immunological diseases are mostly used to treat rheumatoid arthritis and lupus myelopathy. Rituximab which is indicated in lupus myelopathy has gained a great success in clinical application and it is now being explored further for any new indications. Although mAbs are not considered as the drug of choice in ophthalmology, they may possibly become the innovative treatment for neovascular glaucoma and diabetic macular edema as shown in Table 3.

The following examples are used to provide a holistic view of mAbs research development in China. BioTech Pharmaceutical funded the Cancer Center in the Radiology Department of Sun Yat-Sen University to conduct open and multicenter clinical research on the efficacy of cis-platinum combined with Nimotuzumab as the first line treatment of NPC. This clinical research will help shed light on the possibility of “Taixinsheng” combination therapy. Similarly, Chengdu Huasun Bio-Tech funded Fudan University to conduct intervention study to improve clinical outcomes of the combination therapy of “Licartin” and transcatheter arterial chemoembolization (TACE). These involve eight clinical trials which evaluate the clinical effectiveness of mAbs in combination therapies. This is an indication that mAbs as part of a combination therapy has become the new and alternative trend in future research.

### 3.4. Analysis of mAbs-Related Publications in China

Academic publications related to mAbs in China were extracted from Thomson Reuters' Web of Science (WOS) database. This is a powerful database which contains bibliographic data for, and citation to, publications in over 10,000 of the world's most important academic journals dating back to 1900. Studies and research about mAbs in China began in the 21st century [36]. Publications related to mAbs between 2000 and 2013 were searched using the following strategies: Topic = (monoclonal antibod ^*^) and Title = (monoclonal antibod ^*^) AND Address = (CHINA NOT HONGKONG NOT TAIWAN NOT MACAU). “mAbs” was used as the keyword in the same way. In the query above, the asterisk (∗) represents any group of characters or no character and the literature type is limited in “Article.”

**Figure 2:**
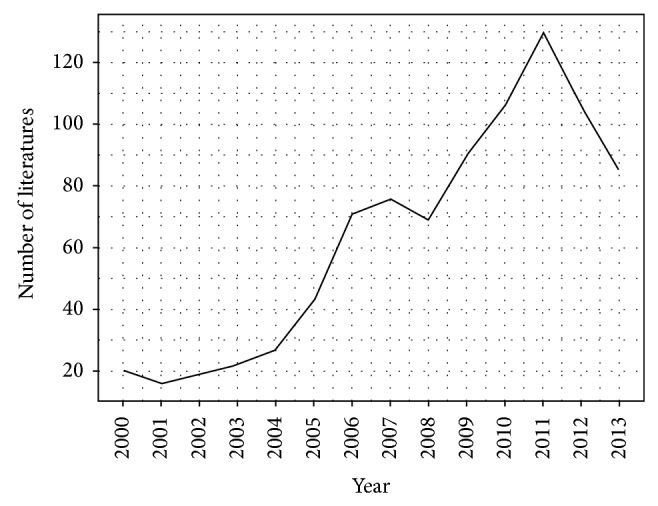
Publication of mAbs in China (2000–2013).

When “monoclonal antibody” was used as the search word, 957 articles were identified. Of these, 788 were retrieved after excluding 169 articles that were irrelevant to our research questions. With “mAb” as acronym words, 114 articles were identified. Of these, 95 articles retrieved after excluding 19 irrelevant records. Finally a total of 878 articles were collected after 5 repetitions of literature search (see Figure 2). The data shows that the number of publications was the most abundant in the last five years (2008–2013) and peaked in 2011, while the decrease in 2012 and 2013 implies the more challenges for Chinese researchers to make breakthrough in more innovative mAb research. This historical trend of research corresponded with the trends of mAbs technology development in China. By using “Result Analysis,” research areas of mAbs in China focused on biochemistry, molecular biology, immunology, biotechnology, applied microbiology, biochemical research methods, chemistry, chemical analytical, and chemistry applied. The data also showed that research in joint forces with international counterparts was a common practice in mainland China especially with the US.

### 3.5. Current mAbs-Related R&D Projects in China

This paper described the development and achievement of research in four main areas, namely, approved products, patent, clinical trials, and publications. In addition, research results also showed that academic institutes and enterprises play an increasingly important role in mAbs research in China. The leading R&D institutions in China are listed in Table 4.

#### 3.5.1. Academic R&D

The R&D institution network in Beijing is mainly associated with military background, enabling access to the upstream or moderate advancement in antibody technologies, such as Academy of Military Medical Science, Institute of Genetics and Developmental Biology, and the Fourth Military Medical University. The research and development in the area of tumor therapy is their core research objective. The success of R&D in this area relies heavily on government policies encouraging ethical achievement of gene cloning, humanized construction [37], high expression, mAbs purification [38], and platform construction of phage antibody library technology [39, 40]. As fully human mAbs are considered the most promising category of targeted therapeutic agents [41], China has also shown great interests. Academy of Military Medical Science, for instance, is dedicated to advancing the screening techniques of phage antibody libraries and optimization platform establishment necessary for the development of fully human mAbs [42].

#### 3.5.2. Industrial R&D

Drug companies in China also play an important role in the development of mAbs, especially in biosimilars [43]. Being one of the representatives among the numerous creative and energetic mAb enterprises in China, BioTech Pharmaceutical developed and produced the first humanized mAb [44]. BioTech also formed collaboration with Cuba Center of Molecular Immunology, which is yet another example demonstrating the strong interests in international cooperation by Chinese enterprises.

The R&D institution network in Shanghai focused mainly on mAbs technology industrialization. One of the R&D companies in the Zhangjiang Hi-Tech Park is Shanghai CP Guojian Pharmaceutical Co., Ltd. (CPGJ) which was founded by China International Trust and Investment Corporation (CITIC) and is now coinvested by Shanghai Lansheng Guojian Pharmaceutical Co., Ltd. Contributions have been made to the R&D, pilot plant test, and industrialization of antibody based drugs like CPGJ. There are other R&D companies in the park which focus on R&D, manufacturing and marketing of high quality recombinant protein for the treatment of immune disease and neoplastic disease such as Shanghai Celgen Biopharma.

## 4. Conclusion and Perspectives

To summarize, mAbs play an important role as efficient agent for antitumor and immunology disease. Eight products in total are launched by CFDA currently, mainly are in chimeric and humanized types. The number of total publications has been increasing since 21st century. Nevertheless higher quality articles are needed. Concerned about the intellectual property, patents applied by Chinese assignee grow rapidly. To monitor safety and effectiveness of mAbs, enterprises give support to and fund clinical trials for antitumor and other new indications. The future for mAbs in China is promising. It should be mentioned that antibody-drug conjugates are becoming an increasingly important subclass of antibody-related cancer therapeutics and glycoengineering is being developed as a method to enhance the pharmacological properties of mAbs. China may also need to pay much more attention to these kinds of antibodies.

This paper found that mAbs have experienced the fastest growth among all human therapeutic agents in the past two decades and will continue to do so in coming years [45, 46] and presented a lot of advancements which have been achieved in China [47, 48]. Chinese biosimilar antibodies may now be approved in Europe [49]; at the same time, some of the most successful innovated biopharmaceuticals currently in the market (including recombinant insulin, human growth hormone, etc.) will see the expiration of their patents in the US over the next few years. This will provide an opportunistic market share which potentially worth 40 billion dollars for biosimilars including mAbs in China [47].

However, there are still some challenges yet to be resolved and at the top of the list would be production capability for China [50]. Firstly, there is still a lack of capacity for large-scale cell and perfusion cultivation at present [51]. To address this problem, improving the cells expression is necessary. China should attempt to work with developed countries because, comparing patent information related to US, the patents applied in both countries mainly involve three of these eight sections (section C, section A, and section G) and, as indicated by the patent search results, the mAbs research in the US encompasses a lot more fields than in China [52], especially in protein expression. Moreover, according to the data searched in WOS, US has been the strategic partner to China in R&D, which as mentioned before indicates that China should be more active to track first-edge technological research to make up the lack of capacity.

Additionally, data from IMS showed that in 2007 mAb drugs accounted for 34.4% of the worldwide total sale, but only 1.7% in China [53], which was far below the global average [16]. While limited by production capability, high price, and low recognition, mAbs in Chinese market perform not well as other countries. To change the status, actions should be taken by the government. The Chinese government is particularly supportive to universities and enterprises by ensuring reliable sources of funding for biopharmaceutical research and development. Some of the most important funding sources are National Natural Science Foundation of China, National High Technology Research and Developmental Program of China, and National Basic Research Program of China. As universities and institutions account for a significant proportion of patent applications, China is steering towards a research-oriented country in the area of mAb development. Accordingly, the government has adopted favorable policies to encourage university-industry cooperation [54]. The government also provides financial support to create and maintain a healthy environment for the biopharmaceutical industry. The inclusion of cancer treatment into the National Social Security would provide prosperous future for mAbs development in China once approved [17]. Inclusion to the medical insurance catalogues also provides another important incentive for thriving mAbs development. For example, basiliximab is listed in the medical insurance catalogues in thirteen provinces while infliximab in nine provinces. Last, measures should also be made to attract much more foreign talents to join in the R&D and benefit from technology around the world. With all of these government support and joint efforts of academic and industry, there the mAb development in China may contribute to global mAbs production and therapeutic innovation. Indeed, we strongly believe that in the near future, there will be likely a rapid growth in the development and usage of mAbs in China owing to the sustained support from government.

## Figures and Tables

**Table 1:** Launched mAbs products in China.

Number	Generic	Trade	Company	Year	Therapeutic category	Target	Type	R&D	Technology source
1	Murine mAb against Human CD3 Antigen of T Lymphocyte Injection	N/A	WIBP	1999	Treatment and prevention for acute rejection of patients received kidney and organ transplant	CD3	Murine	Me-too	Internal R&D

2	Murine mAb against Human Interleukin-8 Cream	Enboke	Asia Space	2003	For the treatment of psoriasis vulgaris and eczema	IL-8	Murine	Me-too	Anogen-Yes, CA

3	Iodine (131I) metuximab injection	Licartin	Chengdu Huasun	2006	For the treatment of primary liver cancer can not be resected with surgery or primary liver cancer relapse and advanced liver cancer not suitable for TACE treatment or ineffective after TACE treatment	CD147	Murine	Me-too	Fourth Military Medical University, CN

4	Iodine (131I) tumor necrosis therapy mAb injection	Weimeisheng	Shanghai Mei-En	2006	For the radio immunotherapy treatment of advanced lung cancer can not be controlled by radioactive chemotherapy or advanced lung cancer relapse	Intracellular DNA	Chimeric	Me-better	University Of Southern California (USC), USA

5	Nimotuzumab injection	Taixinsheng	BioTech	2008	For the radiotherapy treatment of III/IV period of nasopharyngeal with positive expression of epidermal growth factor receptor	EGFR	Humanized	Me-better	Center of Molecular Immunology (CIM), CU

6	Recombinant Humanized Anti-CD25 mAb Injection	Jiannipai	CPGJ	2011	For the prevention of acute rejection after kidney transparent	CD25	Humanized	Me-better	Second Military Medical University, CN

7	Recombinant Human Tumor Necrosis Factor-*α* Receptor II: IgG Fc Fusion Protein Injection	Yisaipu	CPGJ	2005	(1) For the treatment of moderate and severe active rheumatoid arthritis (2) For the treatment of moderate and severe plaque psoriasis patients aged at least 18 (3) For the treatment of active ankylosing spondylitis.	TNF	Recombinant fusion protein	Me-better	Self-development

8	Recombinant Human TNF Receptor-IgG Fusion Protein Injection	Qiangke	Shanghai Celgen	2011	(1) For the treatment of moderate and severe active rheumatoid arthritis (2) For the treatment of moderate and severe plaque psoriasis patients aged at least 18 (3) For the treatment of active ankylosing spondylitis	TNF	Recombinant fusion protein	Me-too	Input from US

Notes: each row of the table represents kinds of information about mAbs products approved in China. “Trade” is brand name of drug selling in Chinese market.

“R&D Type” means that medicines are classified according to different technology and skill levels. “WIBP” is Wuhan Institute of Biological Products, Ltd.

“CPGJ” is Shanghai CP Guojian Pharmaceutical Co., Ltd.

Source: http://www.sda.gov.cn/WS01/CL0001/; author's analysis.

**Table 2:** mAb patents of China in USPTO.

Number	Title	IPC	Assignee	Publication date (MM/DD/YYYY)
1	Use of DKK-1 protein in the cancer diagnosis	A61B 5/00 (20060101); A61B 8/00 (20060101); A61B 10/00 (20060101)	Shanghai Cancer Institute (Shenzhen, CN)	12/27/2012

2	Monoclonal antibodies binding to avian influenza virus subtype H5 hemagglutinin and uses thereof	A61K 39/42 (20060101)	Xiamen University (Fujian Province, CN)	12/22/2011

3	Fully human anti-TNF-alpha monoclonal antibody, preparation method, and use thereof	A61K 39/395 (20060101); C07K 16/24 (20060101); C12N 5/10 (20060101); C12N 15/13 (20060101); C12N 15/00 (20060101)	Shanghai Biomabs Pharmaceuticals Co., Ltd. (Shanghai, CN)	12/06/2012

4	Anti-EFGRv3 monoclonal antibody	A61K 39/395 (20060101); C12P 19/34 (20060101)	Shanghai Cancer Institute (Shanghai, CN)	02/14/2013

5	Use of DKK-1 protein in the cancer diagnosis	A61K 51/00 (20060101); A61M 36/14 (20060101)	Shanghai Cancer Institute (Shanghai, CN)	05/14/2009

6	Anti-VEGFR monoclonal antibody, method of making, and uses thereof	A61K 39/395 (20060101); C07K 16/00 (20060101); A61K 39/00 (20060101); C07K 16/28 (20060101)	Shanghai Aosaiersi Biotech Co., Ltd. (Shanghai, CN)	02/17/2011

7	Anti-human trail receptor DR5 monoclonal antibody (AD5-10), method thereof, and use of the same	C12P 21/08 (20060101); C12N 5/16 (20100101); C12N 5/07 (20100101); A61K39/395 (20060101); C07K 16/00 (20060101)	The Institute of Basic Medical Sciences of Chinese Academy of Medical Sciences (Beijing, CN)	04/01/2010

8	Monoclonal antibody against hepatitis E virus or its fragment with binding activity and use thereof	C12P 21/08 (20060101); C07K 16/00 (20060101)	Beijing Wantai Biological Pharmacy Enterprise Co., Ltd. (Haikou, CN)	10/19/2006

9	Method and composition for diagnosis of melanocytic lesions	G01N 33/53 (20060101); G01N 33/574 (20060101)	Shanghai CP GuoJian Pharmaceutical Co., Ltd. (Shanghai, CN)	N/A

Notes: “IPC” is International Patent Classification. “Assignee” shows the contents of mAb patents belonging to Chinese applicants in United States Patent and Trade mark Office (USPTO).

Source: http://www.uspto.gov/.

**Table 3:** Clinical trial of mAbs in China.

Number	Registry number and scientific title	mAb	Year	Applicant's institution	Funding	Target disease	Study phase	Drug combination
1	(ChiCTR-ONC-13003646) Function of ranibizumab intraocular injection in the control of neovascular glaucoma	Ranibizumab	2013	Qilu Hospital of Shandong University	①	Neovascular glaucoma	Post-market	No

2	(ChiCTR-RCS-13003158) VEGF gene polymorphisms predict of bevacizumab in the treatment of advanced breast cancer research	Bevacizumab	2013	South China University of Technology	Chinese Anti-Cancer Association	Breast cancer	Phase II	No

3	(ChiCTR-ONC-12002442) Clinical study of chemoimmunotherapy with fresh frozen plasma, high dose methylprednisolone, and rituximab for ultra-high risk chronic lymphocytic leukemia	Rituximab	2012	The First Affiliated Hospital of Nanjing Medical University, Jiangsu Province Hospital	Investigator sponsored	Ultra-high risk chronic lymphocytic leukemia	Phase III	No

4	(ChiCTR-ONRC-12002397) Neoadjuvant chemotherapy of bevacizumab, gemcitabine, and cisplatin in stage III NSCLC	Bevacizumab	2012	The First Affiliated Hospital of Guangzhou	N/A	NSCLC	Phase II	Yes

5	(ChiCTR-ONC-12002130) Open-Label, uncontrolled, multicenter phase II study of cisplatin and 5-Fu combined with nimotuzumab as first-line treatment in patients with untreated metastatic nasopharyngeal carcinoma	Nimotuzumab	2012	Sun Yat-Sen University	Biotech	Metastatic nasopharyngeal carcinoma	Phase II	Yes

6	(ChiCTR-TRC-11001556) Optimization treated clinical research for I-131 metuximab injection combined with chemoembolization for HCC	Metuximab	2011	Chengdu Huasun	Chengdu Huasun	HCC	Post-market	Yes

7	(ChiCTR-TNC-11001145) A phase II study of neoadjuvant chemotherapy and IMRT combined with cetuximab in advanced T stage of nasopharyngeal carcinoma	Cetuximab	2011	Sun Yat-Sen University	Merck	Nasopharyngeal carcinoma	Phase II	Yes

8	(ChiCTR-TRC-10001060) Efficacy and safety of infliximab in Chinese patients with rheumatoid arthritis: double-blind, randomized, placebo-controlled trial	Infliximab	2010	Shanghai Changzheng Hospital	CPGJ	Rheumatoid arthritis	Phase III	No

9	(ChiCTR-TNC-10000798) Rituximab in the treatment of severe lupus myelopathy: a prospective, open-label pilot trial	Rituximab	2010	Shanghai JiaoTong University, Renji Hospital	Shanghai Jiaotong University, Renji Hospital	Lupus myelopathy	Post-market	Yes

10	(ChiCTR-TNC-09000606) Triple therapy for diabetic macular edema: intravitreal bevacizumab, triamcinolone acetonide, and macular photocoagulation	Bevacizumab	2010	N/A	Hospital of Talmologico de Sorocaba	Diabetic macular edema	Phase III	Yes

11	(ChiCTR-TNC-08000116) Open, multicenter study of cetuximab combined with IMRT and concurrent chemotherapy of cisplatin in nasopharyngeal carcinoma	Cetuximab	2008	Sun Yat-sen University	Merck	Nasopharyngeal carcinoma	Post-market	Yes

12	(ChiCTR-TRC-08000108) Clinical study of MabThera (Rituximab) and chemotherapy for the treatment in NHL	Rituximab	2008	Fujian Tumor Hospital	Self-supported	NHL	Post-market	Yes

Notes: “Primary Sponsor” is the experiment contract organization, “Funding” represents the resource of fund, from which institutions or others. “①” is The Independent Innovation Foundation to Universities and Colleges by Jinan Science and Technology Bureau. “NSCLC” is “non-small-cell lung cancer.” “NHL” is short for non-Hodgkin's lymphoma. “HCC” is Hepatocellular Carcinoma. “CPGJ” is Shanghai CP Guojian Pharmaceutical Co., Ltd.

Source: http://www.chictr.org/en/.

**Table 4:** Chinese leading mAbs R&D institutions.

Number	R&D institutions	Ongoing	Products	Remarks
1	Academy of Military Medical Science	Gene cloning, humanized construction	Anti-tetanus antibody	At present the only state-level antibody pharmaceutical engineering research institute in China

2	Institute of Genetics and Developmental Biology, CAS	High expression and platform construction of phage antibody library technology, group sequencing to gene mapping and cloning	Antiencephalitis B mAb	N/A

3	Cancer Institute and Hospital, Chinese Academy of Medical Sciences	Humanized engineering and high expression, proteomic study of “gene-environment interactions” of esophagus cancer	N/A	Set the tumor medical research and teaching as a whole

4	Institute of Medical Biotechnology Academy of Medical Sciences and Peking Union Medical College	Molecular miniaturization and high efficient, genetic engineering fusion protein based on Lidamycin and Fab', Fab, VH, VL	N/A	N/A

5	Shanghai Institutes for Biological Sciences, CAS	The introduction and imitation of antibody, preparation of antibody, and proteome research platform support	N/A	Shanghai R&D Public Service Platform

6	Institute Pasteur of Shanghai CAS	Gene cloning, tumor immune cure, and gene therapy which based on DC	N/A	N/A

7	Southern Medical University (former the First Military Medical University)	Tumor targeted biological treatment, applied gene chip	N/A	The first biotechnology experimental base in Guangdong province

8	Cell Engineering Research Center, the Fourth Military Medical University	Screening techniques of phage antibody libraries and optimization platform establishment	Iodine (131I) antitumor necrosis therapy injection	National cell engineering Intermediate test base, antibodies to large-scale preparation technology platform

Notes: each row of the table shows the current status of R&D in China: “Ongoing” represents research activities of the institutions focusing on mAbs. “CAS” is Chinese Academy of Sciences. “DC” is dendritic cell.

Source: author's analysis and online source.
